# Mal de debarquement syndrome: a systematic review

**DOI:** 10.1007/s00415-015-7962-6

**Published:** 2015-11-11

**Authors:** Angelique Van Ombergen, Vincent Van Rompaey, Leen K. Maes, Paul H. Van de Heyning, Floris L. Wuyts

**Affiliations:** Antwerp University Research Centre for Equilibrium and Aerospace (AUREA), University of Antwerp, Groenenborgerlaan 171, 2020 Antwerp, Belgium; Faculty of Medicine and Health Sciences, University of Antwerp, Antwerp, Belgium; Faculty of Sciences, Department of Physics, University of Antwerp, Antwerp, Belgium; Department of Otorhinolaryngology, Antwerp University Hospital, Wilrijkstraat 10, Edegem (Antwerp), Belgium; Faculty of Medicine and Health Sciences, Department of Speech, Language and Hearing Sciences, Ghent University, Ghent, Belgium

**Keywords:** Mal de debarquement, Sea legs, Mal de debarquement syndrome, Systematic review

## Abstract

Mal de debarquement (MdD) is a subjective perception of self-motion after exposure to passive motion, in most cases sea travel, hence the name. Mal de debarquement occurs quite frequently in otherwise healthy individuals for a short period of time (several hours). However, in some people symptoms remain for a longer period of time or even persist and this is then called mal de debarquement syndrome (MdDS). The underlying pathogenesis is poorly understood and therefore, treatment options are limited. In general, limited studies have focused on the topic, but the past few years more and more interest has been attributed to MdDS and its facets, which is reflected by an increasing number of papers. Till date, some interesting reviews on the topic have been published, but a systematic review of the literature is lacking and could help to address the shortcomings and flaws of the current literature. We here present a systematic review of MdD(S) based on a systematic search of medical databases employing predefined criteria, using the terms “mal de debarquement” and “sea legs”. Based on this, we suggest a list of criteria that could aid healthcare professionals in the diagnosis of MdDS. Further research needs to address the blank gaps by addressing how prevalent MdD(S) really is, by digging deeper into the underlying pathophysiology and setting up prospective, randomized placebo-controlled studies to evaluate the effectiveness of possible treatment strategies.

## Introduction

The first real recognition of mal de debarquement (MdD) as a clinical syndrome only occurred in 1987 by Brown and Baloh [1], preceded by allusions made by Darwin and Irwin [2, 3].

Mal de debarquement, also known as ‘sea legs’ [4], rocking dizziness [5] or mal de debarquement syndrome (MdDS) [1, 6], is a subjective perception of self-motion after exposure to passive motion and can sometimes be accompanied by actual postural disturbances. In most cases, MdD (which freely translates to “sickness after disembarkment”) occurs after sea travel; however, MdD can also occur after air or land travel [7, 8]. The underlying pathogenesis of MdD is unclear and it is considered a rare disease [1, 9–11]. In any case, it is important to make a distinction between transient MdD symptoms (<48 h) [12] and persistent MdDS (>3 days up to several years) [1, 13], as the latter is pathological, while transient MdD is a common phenomenon (e.g., in naval personnel) and occurs frequently with reported numbers between 72 and 80 % [4, 12, 14, 15].

Clinically, patients experience a rocking, bobbing or swaying sensation which is often accompanied by unsteadiness and disequilibrium that occurs persistently after cessation of the exposed passive motion stimulus [1, 16]. A high association of MdDS and headache [17] and migraine [13] has been postulated, especially in patients who develop spontaneous MdDS episodes [13]. Previous studies also found associations between MdDS and motion sickness [4], [13, 18], increased self-motion sensitivity [13] and increased visual sensitivity [13]. Consequently, MdDS is associated with a lower quality of life (QoL), higher anxiety and depression rates and has a significant socio-economic impact [19–21]. In general, no structural abnormalities are found on standard brain magnetic resonance imaging (MRI) scans and inner ear function tests are normal [1] and therefore, this is often used as an inclusion criterion in MdDS studies [22–24]. There is a well-documented female preponderance for MdD [1, 13, 25].

Treatment options are limited, but some pharmaceutical agents (e.g., benzodiazepines, selective serotonin reuptake inhibitors) [13, 25], stress relievement therapy [13] and vestibular rehabilitation have been reported as being beneficial [1, 9, 13, 25]. More recently, promising results have been achieved by means of neuromodulation [22, 24, 26, 27] (i.e., repetitive transcranial magnetic stimulation (rTMS)) and modulation of the vestibulo-ocular reflex (VOR) [28], which is assumed to be maladapted in MdDS [29, 30].

Over the past decades, more studies were dedicated to MdDS and its clinical representation [1, 5, 9, 13, 16, 19, 25], socio-economic impact [19–21], underlying pathophysiology [19, 23, 31–33] and possible treatment strategies [18, 22, 24, 28]. Unfortunately, the literature is still scarce and suffers from the lack of generalization due to case reports, small-sample studies and the absence of case–control and placebo-controlled studies. Although interesting reviews on the topic are available [6, 8, 34], a systematic review on existing MdD and MdDS literature is lacking. A thorough synthesis could help to efficiently set-up and conduct future research on the topic.

The aim of the present study was twofold: (a) to conduct a systematic review of studies describing the epidemiology, diagnostic procedure, the (neuro)pathophysiology and treatment options of this rare and poorly understood entity and (b) to identify flaws in the existing literature concerning mal de debarquement and concurrently summarize future research opportunities.

## Materials and methods

### Search strategy

The Medline (PubMed) and EMBASE databases were searched for papers using the term “mal de debarquement” and “sea legs” without restriction of publication date. Reference lists from retrieved articles were also searched manually for relevant publications on either mal de debarquement that were not included in the lists created through the Medline database. Research abstracts from meeting proceedings or unpublished studies were not included. The search was last updated on 18 September 2015.

The title and abstract of all of the articles yielded by the search were screened by two independent reviewers and selected using predetermined criteria. Non-English studies and case and anecdotal reports were excluded. Other exclusion criteria were a lack of original patient data (e.g., reviews) or duplications of data published in other included papers.

Full-text screening was applied to all abstracts considered eligible by at least one reviewer.

### Data extraction and analysis

A table (Table 1) was constructed to summarize relevant results from the selected studies. Study designs, objectives and outcome measurements were discrepant and not amenable to quantitative analysis. Data were classified and analyzed qualitatively.

**Table 1:** Qualitative synthesis on the demographic features and inclusion criteria of the included MdD(S) studies

Study	Pro-(p) or retrospective (r)	Healthy individuals (h)/patients (p)	Subjects (*n*)	M/F	Mean age (SD) (years)	Inclusion criteria
Brown and Baloh [1]	r	p	6	1/5	50.8 (14.3)°	nc
Gordon et al. [13]	r	h	234	234/0	20.5 (nd)	n/a
Murphy [8]	r	p	4	0/4	nd	nc
Gordon et al. [11]	r	h	116	116/0	nd	n/a
Cohen [14]	r	h	59	nd	nd	n/a
Hain et al. [24]	r	p	27	1/26	49.3 (10.3)	(a) Diagnosis of MdDS by at least 1 physician
						(b) Sensation of rocking or swaying that persisted at least 1 month following a 4-h or longer exposure to motion on an airplane or boat
Nachum et al. [15]	p	h	34	34/0	nd, age range: (18–22)	n/a
Cha et al. [12]	r	p	64	16/48	nd	Internal sensation of motion such as rocking, swaying or bobbing lasting at least 3 days after exposure to passive motion
Gibbs et al. [35]	p	h	39	nd	nd	n/a
Macke et al. [19]	r	p	101	3/98	52.0 (10.9)	(a) Self-reported diagnosis by a licensed physician
						(b) Experiencing MdDS symptoms for at least 4 weeks
Cha et al. [22]	p	p	20	5/15	43.4 (nd)	(a) Chronic perception of rocking dizziness that started after passive motion such as sea, air or land travel
						(b) Symptoms lasted at least 1 month
						(c) Normal inner ear function testing with ENG/VNG and audiograms
						(d) Normal structural brain imaging with a non-contrast MRI
						(e) First lifetime episode of MdDS
						(f) No other cause of symptoms after evaluation by a neurologist
Cha et al. [36]	p	p	10	2/8	nd, age range: (27–59)	(a) Chronic perception of rocking dizziness that started after disembarking from sea, air, or land-based travel
						(b) Symptoms lasted at least 6 months
						(c) Normal peripheral inner ear function testing with ENG/VNG and audiograms
						(d) Normal structural imaging with brain MRI
						(e) No other cause of symptoms after evaluation by a neurologist
Clark et al. [18]	p	p	8	0/8	47.5 (15.2)	(a) Diagnosed with MdDS by a neurologist or otolaryngologist
						(b) Experiencing an active episode of persistent MdDS that was triggered by passive motion for the past 60 days that were initially triggered by passive motion
Cha et al. [16]	p	p	76	14/62	nd; median: 45, age range: (12–69)	(a) Primary symptom of chronic rocking dizziness lasting at least 1 month
						(b) Symptoms must have occurred within 2 days of disembarking from a boat, airplane or land vessel with motion exposure lasting at least two continuous hours
						(c) Normal peripheral vestibular function testing with either ENG or VNG
						(d) No other cause for rocking dizziness after evaluation by a neurologist or otolaryngologist after appropriate testing
Stroffregen et al. [3]	p	h	24	4/20	20.5 (2.3)	n/a
Tal et al. [17]	p	h	30	nd	nd	n/a
Dai et al. [27]	p	p	24	3/21	43.0 (8.8)	(a) Continuous rocking, swaying and/or bobbing that began shortly after exposure to a voyage on water or in the air and that persisted for months or years
						(b) Symptoms were debilitating
						(c) Symptoms could not be relieved by medication or other medical treatments
						(d) Symptoms were temporarily better during car rides or travel on water but returned after the rides were terminated
Arroll et al. [20]	r	p	66	4/62	52.1 (12.2)	Patients were self-selected
Pearce et al. [21]	p	p	14	6/8	63.5 (12.6)	(a) Diagnosis of MdDS and referral from their neurologist based on criteria by Cha et al. [36]
Cha et al. [30]	p	p	29	5/24	43.0 (10.2)	(a) A typical history of chronic rocking dizziness occurring within 2 days of disembarking from a moving vessel such as from sea, air or land-based travel
						(b) Symptoms lasting at least 3 months without any other cause found after evaluation by a neurologist or otolaryngologist
						(c) Normotensive (for neuro-imaging study)
						(d) Pass a screening neurological examination

*n/a* not applicable, *nc* not clear, *nd* not described, ° self-calculated by data presented in study

Ethics committee authorization was not required as this study reviewed previously published data.

## Results

The database search yielded 48 citations and hand search added 5 articles. The oldest article was published in 1987 by Brown and Baloh [1] and the most recent was published in 2015 by Nwagwu and colleagues [35]. Full-text review resulted in the exclusion of 34 articles, resulting in 19 eligible articles. Seven articles reported findings related to MdD from a healthy population (i.e., crew members of seagoing vessels [12, 14, 16], sailors [15], staff and passengers on a dive trip [36], students taking part in an education program at sea [4] and healthy participants exposed to a ship motion simulator [18] ), while the other 12 articles report surveys and experiments in patients [1, 13, 17, 19–23, 25, 28, 31, 37]. A summary of the articles that were selected for inclusion can be found in Table 1. A qualitative synthesis and critical appraisal of the patient-related studies can be found in Table 2.

**Table 2:** Qualitative synthesis and critical appraisal of the MdDS patient-related studies

Study	Objective(s)	Outcome measure(s)	Main finding(s)	Main limitation(s)	Evidence^Ψ^
Hain et al. [24]	To define MdDS.	Clinical features of MdDS	MdDS usually occurs in middle-age women	Patients recruited from a “dizzy population”	3
				No control group	
			MdDS is usually preceded by an ocean cruise		
			Symptoms are often refractory to vestibular suppressant and physical therapy		
	To understand etiology or underlying mechanism				
		DHI scores			
	To investigate prevention and treatment options				
Cha et al. [12]	To investigate the clinical features, associated syndromes and natural history of MdDS	Clinical features of MdDS	An MdDS patient is an otherwise healthy individual who develops a perception of rocking or swaying after a period of passive movement, obtains relief with re-exposure to passive movement and has normal diagnostic testing including vestibular and brain imaging	Long study span (26 years)	3
		ENG and MRI results		No control group	
		Questionnaire responses			
			Majority of MdDS episodes lasting longer than 3 days resolve in less than 1 year		
			Majority of MdDS patients experience multiple episodes		
			Migraine is a risk-factor to develop spontaneous MdDS episodes		
Macke et al. [19]	To investigate the impact of MdDS on QoL and to estimate the economic costs associated with MdDS	Adapted version of MSQOL-54°	MdDS negatively impacts QoL	Lack of control group	3
			MdDS imposes a substantial economic burden		
		Direct economic costs		Self-reported patients	
				No follow-up	
Cha et al. [22]	To investigate if MdDS is reflected by changes in brain metabolism and functional connectivity in areas that process and store spatial information	Gray matter volume	Hypermetabolism in left EG and amygdala	Controls not matched for motion exposure	2
		Brain metabolism	Hypometabolism in prefrontal and temporal lobe		
		Functional connectivity			
			Increased functional connectivity in posterior spatial processing areas		
			Decreased functional connectivity in prefrontal areas		
Cha et al. [36]	To investigate the feasibility, tolerability, side effects and possible therapeutic effects of rTMS in MdDS	Symptom severity on VAS	Short-term improvement of rTMS on MdD symptoms	Relative small sample size	2
		Edinburgh handedness inventory	Minimal side effects of rTMS	No formal sham condition	
			Handedness seems to be related to MdD physiology		
Clark et al. [18]	To investigate if persistent MdDS patients exhibited differences in intracortical and corticospinal excitability	CoP measurements.	MdDS patients exhibit impaired postural control	Small sample size	2
		MEP and MT	MdDS patients exhibit high levels of kinesiophobia and fatigue		
		Score on Center for Epidemiologic Studies Depression Scale°			
			MdDS patients exhibit higher MT and large MEP amplitude, i.e., increased corticospinal excitability		
	To characterize postural control and psychological impact of MdDS	Score on functional assessment of chronic illness therapy—fatigue° survey	No differences in measures of intracortical excitability		
		Score on tampa scale for kinesiophobia°			
Cha et al. [16]	To clarify the association between motion-triggered and non-motion triggered chronic rocking dizziness and headache history	Questionnaire			3
		Interview			
Dai et al. [27]	To investigate the therapeutic effect of remodulation of the VOR on MdDS symptoms	Subjective symptom severity (number from 0 to 10)	Remodulation of the VOR is effective in the majority of MdDS patients	No control group	3
		Postural sway	MdDS will most likely not occur in subjects with very short VOR time constants		
Arroll et al. [20]	To investigate QoL (physical and mental) and illness intrusiveness in MdDS patients	Score on SSCI scale° (stigma-related)	MdDS is associated with high levels of illness intrusiveness, depression and a reduced QoL	Patients were self-reported	3
				Lack of control group	
		Score on IIRS scale° (illness intrusiveness-related)			
	To study the degree of stigma in MdDS patients				
		Score on center for epidemiologic studies depression scale°			
		Score on SF-12 health survey° (QoL-related)			
		Measure of symptom severity			
Pearce et al. [21]	To investigate the beneficial effect of rTMS on MdDS	Score on miniBEST°	Larger effect size of real rTMS pre and post than for sham TMS on miniBEST score	Small sample size	2
		Score on ABC-scale questionnaire°		No reported p-values	
			Larger effect size of real rTMS pre-middle and pre-post than for sham TMS on ABC score		
				Remains speculative about rTMS effect	
Cha et al. [30]	To identify neural substrates underlying MdDS and to investigate the correlation of gray matter volumes with duration of the disease	Grey matter volume	Differences in grey matter in:	No insight into causality versus consequence of grey matter abnormalities in MdDS	2
			Visual-vestibular processing areas		
			Default mode network structures		
			Salience network structures		
			Somatosensory network structures		
			Central executive network structure	Most of the findings were found with uncorrected *p* values	

*ABC-scale* activities-specific balance confidence scale, *CoP* centre of pressure, *DHI* dizziness Handicap inventory, *IIRS* illness intrusiveness ratings scale, *MdDS* mal de debarquement syndrome, *MEP* motor evoked potential, *miniBEST* mini balance evaluation systems test, *MT* motor threshold, *QoL* quality of life, *rTMS* repetitive transcranial magnetic stimulation, *SSCI* stigma scale for chronic illness, *SF-12* short-form health survey, *VAS* visual analogue scale, *VOR* vestibulo-ocular reflex

° References of specific tests or questionnaires can be found in the original articles

^Ψ^Level of evidence according to the Oxford Centre for Evidence-Based Medicine 2009 Levels of Medicine

The percentage of agreement between the two reviewers was high (96.3 %), yielding an interrater agreement kappa coefficient of 0.92 (SE = 0.054).

### Epidemiological and demographic data

MdDS, as seen in its persistent form in patients, is considered a rare disease and prevalence numbers in the general population are yet to be investigated. On the contrary, transient MdD symptoms after exposure to passive motion are common in a normal population with reported numbers of 59 % [18] on a ship motion simulator and up to 72 % [12], 73 % [14], 79 % [4] and even 80 % [15] after actual sea travel.

MdDS has been described by Hain and colleagues as a condition occurring most frequently in middle-age women [25], which is corroborated by the majority of subsequent studies. Cha and Cui reported the peak incidence a bit more specific and reported this as being the fifth life decade [17], which was in accordance to the findings by Arroll et al. reporting a mean age of 52.1 years (SD = 12.2) [21]. Pearce and colleagues reported results in patients with a mean age of 63.5 years (SD = 12.6) [22], but no possible reasons were mentioned for why their sample was considerably older than previously studied patient groups.

Both females and males can suffer from MdDS, however, the sex distribution has been described as predominantly female [1, 9, 13, 17, 19–25, 28, 31]. Numbers of male distribution among MdDS patients vary between 0 and 25 % (Table 1).

No studies have reported on ethnic distribution so far, apart from Hain and colleagues describing 92.6 % of their patients’ population as Caucasian and 7.4 % as Hispanic [25].

### Clinical features

Symptoms can be triggered by most forms of passive motion exposure, nonetheless, sea travel (e.g., boat trips or a cruise) is reported as being the most prevalent with values of 60.6 % [21], 66 % [17], 81 % [13] and 83.3 % [1]. Air travel has been described as a trigger in 41 % [13] or less [1], while land travel (e.g., car or train) has been described in a smaller group of 16 % [13, 21] or less [17]. Anecdotally, patients also reported MdDS occurring after funfair rides and playing motion games on a Nintendo^®^ Wii [21]. One study divided patients into a “pure” MdDS (only motion-triggered) group and a “mixed” MdDS group (motion-triggered and spontaneous) [13]. Other commonly described non-motion triggers include stress [13, 25], positional changes [25], head movements [25] and hormonal changes [13, 25]. In the study by Hain and colleagues, 80 % of the female subjects were either premenopausal or receiving hormone replacement therapy [25].

Inclusion criteria are not uniformly decided upon, however, there are some similarities among studies to which criteria are used to diagnose MdDS. All of the inclusion criteria used in previous studies, if any, are listed in Table 1.

The most prominent symptoms associated with MdDS are the subjective feeling of rocking, swaying and/or bobbing. Hain and colleagues reported rocking in 93 % of the patients and swaying in 81 % [25]. Other symptoms include disorientation [28], postural instability [16, 19, 28], imbalance [25], fatigue [19], impaired cognition and kinesiophobia [19]. In one study, the following symptoms were also associated with MdDS (in order of how frequently they appeared): ear symptoms (non-specified), tilting, nausea, headache, jumping vision, blurred vision, perioral tingling, spinning (vertigo), diplopia, vomiting, eye twitches, fuzzy-headed/woozy, pulling/numbness in foot or lower leg [25]. It has to be noted here that these are symptoms that MdDS patients reported when filling in questionnaires from a survey in 27 patients. It does not necessarily mean that all these symptoms are associated directly with MdDS. In the same study, a high occurrence of otological symptoms (fullness, tinnitus, hyperacusis, otalgia and decreased hearing) was found [25], which is not in accordance with the findings in other studies [13].

In general, standard brain imaging (MRI) and oto-vestibular tests such as measured by electronystagmography, videonystagmography and audiograms are normal or non-specifically abnormal [1, 13, 25]. A normal neurological and oto-vestibular exam is therefore often used as an inclusion criterion (Table 1. for an overview). A positional nystagmus has been reported in two articles; however, the authors did not attribute this directly to the MdDS [1, 9].

### Associated disorders

Migraine has been reported to be typically associated with MdDS, with an overall prevalence higher than population baseline [13]. In addition, both migraine and MdDS have been shown to have a female preponderance [1, 9, 13, 17, 19–25, 28, 31] and can be triggered by stress and hormonal changes [13, 25]. Nonetheless, this was not supported in the study by Hain and colleagues, where only 22 % of their patients met the migraine criteria [25]. In 2008, it was reported that “pure” MdDS patients do not show a significantly higher prevalence of migraine than the general population. However, “mixed” MdDS (also suffering from spontaneous episodes) showed a very significant higher prevalence of migraine (17 % in the “pure” group against 73 % in the “mixed” group). More recently, Cha and Cui investigated the relationship between migraine and motion-triggered rocking dizziness (as is the case in MdDS) and non-motion-triggered rocking dizziness (i.e., spontaneous) [17]. They found that both groups had a similar prevalence of headache meeting the migraine criteria; however, the non-motion-triggered group was much more likely to have a pre-existing migraine than those with MdDS. Therefore, a possible overlap between the underlying pathogenesis in MdDS and migraine is assumed [17].

The relationship between motion sickness and mal de debarquement was already alluded by Irwin in 1887 in his paper “The pathology of sea-sickness” [2]. This relationship is based on the underlying problem in both entities, namely a maladaptation on land (stable conditions) after an exposure and concurrent adaptation to motion. This association was reported by several studies [4, 12, 14, 18]; however, it cannot be made directly. In addition, it has been proven to be related to the experience at sea; the less someone is familiar with sea travel, the higher one will be susceptible to develop sea sickness and dizziness [12, 14]. Furthermore, as shown by Hain and colleagues in their retrospective analysis, only one-third of the patients who developed motion sickness after sea travel had actually taken anti-emetics to prevent motion sickness on board [25]. Therefore, it can be assumed that motion sickness is not per se a contributing factor to MdDS but the exact relation is to be revealed.

An increased visual sensitivity in MdDS patients has been reported recently [13] and ranges from problems with turning pages to difficulties in complex and challenging visual environments. A possible explanation has been described by Cha et al. and revolves around a probable differential weighting of visual and vestibular information during motion [13]. The exact underlying mechanism is not known and has to be further investigated. In the same study, they did not find an increase in self-motion sensitivity (i.e., motion sickness) [13].

### Psychosocial and economic impact

MdDS is a debilitating condition and therefore, it inevitably has an impact on the psychosocial and economic status of patients suffering from MdDS.

An important factor contributing to the debilitating effect of the disease is the long duration between the start of the symptoms and an actual accurate diagnosis, which can take up to several years [13, 25]. This has also been reported as a catalyst to secondary mood disorders such as depression and anxiety [13].

One study investigated the socio-economic burden in a retrospective analysis in 101 patients and they found that MdDS negatively impacts quality of life (QoL) in these patients and in addition, that it also imposes a significant economic burden [20]. On average, 19 visits to a healthcare professional are necessary before receiving a diagnosis of MdDS [20]. This is consistent with a direct cost of $2997 ± 337 per patient and this does not involve other indirect costs that might be associated, such as for example the loss of an income due to the incapability to work [20]. Up to 31 % of the respondents assessed by Hain reported a change in occupational status due to MdDS [25]. Another retrospective study focused on the stigma and illness intrusiveness of MdDS and they found that MdDS is associated with a high level of intrusiveness as well as reduced QoL [21].

Hain and colleagues also assessed the scores of the Dizziness Handicap Inventory and found an average of 45.6 (SD 20.8) and demonstrated that the DHI score was related to the number of symptoms and to the presence of certain symptoms such as headache and imbalance [25]. The DHI score was also negatively correlated with the disease duration [25].

### Pathophysiology

The underlying pathogenesis in MdD(S) is poorly understood and it has only been since past 3 years that studies were set up to investigate it properly. A hallmark was reached in 2012 by a neuroimaging study by Cha and colleagues [23]. For the first time ever, metabolic and functional connectivity alterations were shown in MdDS patients. This was important, as this could be used as a possible biomarker and therefore, objectify MdDS.

Twenty MdDS patients were scanned by means of ^18^F-fludeoxyglucose positron-emission tomography (PET) to look for differences in brain metabolism between MdDS patients and their healthy age- and gender-matched controls [23]. A hypermetabolism in MdDS patients was found in the left entrorhinal cortex (EC) and amygdala. In parallel, a hypometabolism for MdDS patients was found in areas diffusively spread in cortical and subcortical regions. Additionally, functional MRI also showed an increase in functional connectivity between the EC/amygdala and the visual and vestibular processing areas, whereas a decrease between the EC/amygdala and several prefrontal areas was found in MdDS patients [23].

Even more recently, gray matter alterations have been found in 29 MdDS patients in the visual-vestibular processing areas, default mode network structures, salience network structures and the dorsolateral prefrontal cortex (DLPFC) [31]. The authors relate these findings to the fact that these results could explain some of the clinical features associated with MdDS such as an increased visual sensitivity and rocking dizziness at rest that alleviates when being re-exposed to passive motion [31].

These results are very promising, especially because they can help in the development of treatment protocols, such as neuromodulation techniques.

### Treatment

Information regarding the therapeutic possibilities for MdDS was mentioned in several articles, however, only three studies actually set-up a research design to investigate the effect of a specific approach [22, 24, 28]. The others were restricted to assessment through questionnaires and surveys [13, 25].

The use of pharmaceutical agents is described in several studies. In particular, benzodiazepines and selective serotonin re-uptake inhibitors have been described to modestly alleviate MdDS symptoms [13, 25]. More specifically, it was reported by Hain and colleagues in their survey that clonazepam was helpful in 3 out of 6 patients, diazepam in 2 of 10 and alprazolam in 1 out of 4 [25]. On the contrary, anti-emetics (e.g., scopolamine), vestibular suppressants (e.g., meclizine), beta-blockers, calcium channel clockers, diuretics and anticonvulsants have generally been considered as unhelpful [13, 25]. Diet modifications (e.g., decreasing salt intake) have not shown to be helpful either [13]. Another therapeutic approach that has been suggested to be beneficial is stress reduction [13]. The latter is probably related to the fact that emotional and physical stress are postulated as possible triggers for MdDS symptoms [13, 25] and a high percentage of patients indicated they felt highly stressed [25]. Alleviation of symptoms by physical and vestibular therapy was reported by MdDS patients, which was described by Hain and colleagues in 1999 (10 out of 15 patients reported alleviation) [25] and a decade later by Cha and colleagues in 2008 (15 patients reported on average a “small but noticeable improvement”) [13].

Re-exposure to passive motion has also been described several times to induce a temporary alleviation in MdDS symptoms in up to 80 % [13, 21], [25]. The method of travel that decreases symptoms in the majority of patients is car travel, while lengthy travel (e.g., long haul flight) can worsen symptoms in some patients [21]. Dai and colleagues used alleviation by exposure to passive motion as an inclusion criteria for their investigations in MdDS patients [28].

Based on the recently acquired insights into the underlying neuro-pathophysiology, neuromodulation was lately used in attempts to alleviate MdDS symptoms [22, 24, 27]. A relatively higher brain metabolism was found in MdDS patients in the left DLPFC in comparison to healthy controls [23]. In addition, the DLPFC is known to be involved in the cognitive control of spatial processing functions such as spatial working memory. This technique was first applied by Cha and colleagues in an experiment where they applied high-frequency rTMS over the left DLPFC in 10 patients [24]. Although the results are quite promising, the study suffered from a relatively small sample size and the lack of a control group and/or sham-controlled condition. Beneficial effects were also found by low-frequent rTMS over the ipsilateral DLPFC in half of the patients studied [27]. Pearce and colleagues also investigated the possible effectiveness of rTMS over the DLPFC and they did so by a randomized, sham-controlled study design. They concluded upon a suggestive beneficial effect of rTMS on MdDS symptoms, but could not validate this by formal statistical differences between the real and sham TMS condition. Unfortunately, study homogeneity did not allow proper comparison between the three studies.

Another therapeutic approach that has been published recently is VOR modulation, suggested by Dai and colleagues [28]. In this paradigm, the starting point is the assumption that the VOR is maladapted in MdDS patients, based on animal studies [29] and human space experiments [30]. They implemented a protocol in which 24 MdDS patients were treated by rolling the head from side-to-side (in the pitch and/or roll plane) at the frequency of subject’s rocking, whilst watching a rotating full-field OKN stimulus. This was done in 1–8 treatment sessions, spread over five consecutive days. They found a significant decrease in subjective MdDS symptoms, as well as postural sway. Seventy percent of the patients became asymptomatic after the treatment or had a substantial remission for 4 months or longer. In comparison to the findings in neuromodulation trials, the technique of VOR adaptation seems to be beneficial for the majority of patients with an alleviation of longer duration.

## Discussion

To the best of our knowledge, this is the first systematic review of literature on mal de debarquement.

First, it is important to make a distinction between transient MdD and MdDS. At this point, this is often used in a random fashion in the existing literature and therefore, not always accurate and even confusing. Therefore, we suggest the following: the term ‘mal de debarquement’ (MdD) should be used if it concerns the transient form, which basically is an “aftereffect” of passive motion and commonly occurring. MdD includes symptoms of rocking dizziness and instability after exposure to passive motion, most frequently sea travel. On the contrary, ‘mal de debarquement syndrome’ (MdDS) refers to the pathological entity in which patients experience a set of symptoms after exposure to passive motion for a long period of time (or persistently for that matter), has a wide variety of associated symptoms and in addition, a debilitating effect on QoL of the person in question. This distinction should be used meticulously and consequently in future studies to avoid misunderstanding and misinterpretations.

Additionally, a consensus on the exact duration to distinguish between MdD and MdDS has to be agreed upon. So far, there is no real agreement. This makes it confusing and ambiguous to differentiate between the two. Previous studies by Gordon and colleagues found that MdD symptoms resolved within 48 h in an otherwise healthy population [12, 14]. A more recent study, however, reported MdD symptom resolution within 120 min in a healthy population [4]. Based on the review of the literature, a period of 1 month seems to be on average the best proposition as this is confirmed by several studies [13, 20, 25]. Cha and colleagues even went a step further and included the distinction between ‘normal’ (i.e., MdD), prolonged and persistent symptoms (i.e., MdDS) [13]. The additional ‘prolonged’ category could then be implemented for people in whom it is not clear whether they suffer from the non-pathological MdD or MdDS. They suggest to use ‘prolonged’ for symptoms lasting between 48 h and 1 month. Concurrently, any symptoms lasting longer than 1 month are classified as persistent [13].

As reported in several studies, there is often a long duration between the onset of symptoms and the receiving of a diagnosis. Is it proven that this has a negative impact on QoL [20, 21] and that it can lead to secondary mood disorders such as depression and anxiety [13, 21]. In addition, it might also complicate and compromise the ability to acquire a correct diagnosis as the onset of symptoms, triggers and associated disorders (information that might add to a correct diagnosis) become blurry and indistinguishable from other disorders over the years. Therefore, we strongly urge for the implementation of diagnostic criteria when seeing patients with MdDS-like symptoms. If we look at the inclusion criteria used by the existing studies (listed in Table 1), there is some overlap. In our opinion, the ones presented by Cha and colleagues in the majority of their papers, are the most sufficient and complete. Therefore and based upon the criteria implemented by Cha in several of his papers over the years [5, 13, 23, 24, 34], we suggest the following set of criteria to be used as a guideline for healthcare professionals to whom MdDS patients might consult (e.g., general practitioners, neurologists, otolaryngologist, etc.) in Table 3. This summary might help as a potential guideline to reduce the time to acquire a diagnosis and indirectly, to reduce the socio-economic impact on one’s life. Nevertheless, a consensus is yet to be reached and will require more evidence and research into the complex disorder that is MdDS.

**Table 3:** Diagnostic guidelines for MdDS

(a) Chronic perception of rocking dizziness (e.g., rocking, bobbing, swaying) that started after passive motion such as sea, air and land travel or exposure to virtual reality
(b) Symptoms lasting at least 1 month
(c) Normal inner ear function or non-related abnormalities as seen by ENG/VNG and audiological tests
(d) Normal structural brain imaging or non-specific alterations with a non-contrast MRI scan (when no additional more advanced analyses were carried out)
(e) Symptoms not better accounted for by another diagnosis made by a physician

Concerning the first criterion (a), we added “…or exposure to virtual reality” as it has been reported anecdotally that virtual reality environments and stimulators may also trigger MdD(S) [18]. Further research should provide more evidence into the exact underlying mechanism and whether it is completely identical to actual travel. However, we believe it should not be excluded. Concerning criterion (c), we added “…or non-related abnormalities” because it is evident that a conductive hearing loss for example is not related to MdDS. To criterion (d), we added ‘or non-specific alterations” as it is possible that some patients show incidental abnormalities, not related to the MdDS symptoms. In addition, we also added “… when no additional analyses were carried out” to make sure to nuance between a normal structural brain scan per se and a normal structural brain without further analyses (e.g., voxel-based morphometry [38]). Recent studies have shown that there are indeed grey matter abnormalities (i.e., structural) in MdDS patients when compared to healthy controls [23, 31]. This is also in accordance with preliminary data acquired in our institute (Van Ombergen et al., unpublished data). Criterion (e) is similar to the one described by Cha “No other cause of symptoms after evaluation by a neurologist” [23, 24], although we rephrased it to be in analogy with diagnostic criteria for similar entities, such as vestibular migraine [39]. In addition, we changed neurologist to the more general ‘physician’. Indeed, experts will mostly be neurologists or otolaryngologists; however, we believe it is better not to limit it to neurologists per se.

Dai and colleagues used the criterion “symptoms are debilitating” in their 2014 paper [28]; however, we do not fully concur with this, as this is a subjective perception and therefore can be interpreted differently among individuals and is very individually dependent. Obviously, long-standing symptoms of rocking dizziness and instability will have an effect on QoL for most individuals and if not, they probably will not pursue seeking healthcare in the first place. Therefore, we did not add this criterion to the presented list above, although it can still be verified by the healthcare professional consulting with the patient.

In addition, the degree of MdDS symptoms is currently often estimated using of a clinical scale score (1–10), e.g., by Dai and colleagues [28]. However, it would be interesting to investigate if this scale can be associated with more specific symptoms and severity, as is for example the case for motion sickness with the scale provided by Graybiel and colleagues [40]. This would be beneficial for more accurate clinical assessment and diagnostics.

Another point of disagreement seems to be the duration of which the passive motion has to been experienced. In two studies, a specific requirement towards the duration of passive motion is described; a minimal of 2 h exposure to passive motion [17] and a minimal of 4 h exposure [25]. No further reports have been made and therefore, it is not clear if there is a “minimal load” of passive motion exposure necessary to elicit MdD(S) and whether this is different for MdD symptoms and MdDS.

A small remark that has to be made is the fact that all of the above refer to the typical “motion-triggered” MdDS. However, reports have been made about an entity in which isolated, ‘spontaneous’ episode of MdDS-like symptoms occur [13]. This has not been taken into account in the current presented guidelines for diagnosis in MdDS patients (Table 3), because future prospective studies should shed more insight into this matter first. Presumably, but this has to be defined, these patients belong to a distinctive as these patients show overlap between different pathologies, e.g., migraine [13, 17].

The pathophysiology of MdD(S) has been poorly understood, but recent neuroimaging studies have shed more light and seem promising as they could potentially serve as future biomarkers. Changes in structural, functional and metabolic brain properties have been found [23, 31] and they suggest an underlying neural correlate to MdDS. However, further research should dive deeper into this as the results so far are sometimes non-specific (i.e., in areas that hold a multitude of functions) and do not gain insight into the relation of these brain alteration to MdDS or the causality, i.e., does MdDS develop in individuals with these alterations or do these alterations occur after acquiring MdDS? In addition, they suffer from several limitations, as listed in Table 2 and should therefore be interpreted with some consideration. In the most recent neuroimaging paper on MdDS, the majority of findings was published with an uncorrected *p* value. As a lot of comparisons are made with VBM (i.e., voxel per voxel for the whole brain), a corrected *p* value (e.g., Bonferroni or false discovery rate) must try to overcome any false positive and negative findings. Therefore, it is possible that some of the findings presented are actually false positives. Most of these techniques are advanced and therefore, not always clinically obtainable and in addition, they are quite costly and time-consuming. Nevertheless, it is a first potential biomarker that could objectify MdDS symptoms and studies in line of this are necessary if we want to understand what is causing MdD(S) and how to resolve the symptoms.

Treatment options have been described above and are quite limited. A possible therapeutic approach that has been suggested as being beneficial is stress reduction [13]. This is probably related to the fact that emotional and physical stress are often triggers for MdDS symptoms [13]. Caregivers should keep this in mind, as this does not involve any exclusion criteria (unlike neuromodulation) and is less intrusive. Recently, studies have reported promising effects of neuromodulation on MdDS symptoms. However, the evidence is rather limited as it concerned studies without a sham-control [24] or investigating a small sample size [22]. Prospective, randomized placebo-controlled studies should really try to corroborate the therapeutic options, which are currently based on subjective assessment by the patient and therefore, merely suggestive of being beneficial.

In conclusion, we summarized the main findings and limitations of the existing studies on MdD/MdDS. By doing so, we hope to encourage future investigators to overcome the limitations of the current studies. In addition, we suggested a possible list of diagnostic criteria, which should encourage further discussion among the neuro-otologic community, but could be used as a preliminary guideline for healthcare professionals in diagnosing MdDS patients. Further research needs to address the blank gaps by addressing how prevalent MdDS really is, by digging deeper into the underlying pathophysiology and setting up prospective, randomized placebo-controlled studies to evaluate the effectiveness of possible treatment strategies.

## References

[1] Brown J, Baloh R (1987). Persistent mal de debarquement syndrome: a motion-induced subjective disorder of balance. Am J Otolaryngol.

[2] Irwin J (1881). The pathology of sea-sickness. Lancet.

[3] Darwin E (1796). Why after a voyage ideas of vibratory motions are perceived on shore.

[4] Stoffregen TA, Chen FC, Varlet M, Alcantara C, Bardy BG (2013). Getting your sea legs. PLoS One.

[5] Cha Y-H, Cui Y (2013). Rocking dizziness and headache: a two-way street. Cephalalgia.

[6] Parker D, Jennings S (2008). Mal de debarquement syndrome: review of an unusual cause of dizziness. Audiol Med.

[7] Lewis R (2004). Frequency-specific mal de debarquement. Neurology.

[8] Cha Y (2009). Mal de debarquement. Semin Neurol.

[9] Murphy TP (1993) Mal de debarquement syndrome: a forgotten entity?. 109(1): 10–13 10.1177/019459989310900103 8336953

[10] Mair I (1996). The mal de debarquement syndrome. J Audiol Med.

[11] Gordon C, Shupak A, Nachum Z (2000). Mal de debarquement. Arch Otolaryngol Head Neck Surg.

[12] Gordon C, Spitzer O, Doweck I, Melamed Y, Shupak A (1995). Clinical features of mal de debarquement: adaptation and habituation to sea conditions. J Vestib Res.

[13] Cha Y, Brodsky J, Ishiyama G, Sabatti C, Baloh R (2008). Clinical features and associated syndromes of mal de debarquement. J Neurol.

[14] Gordon C, Spitzer O, Shupak A, Doweck I (1992). Survey of mal de debarquement. BMJ.

[15] Cohen H (1996). Mild mal de debarquement after sailing. Ann N Y Acad Sci.

[16] Nachum Z, Shupak A, Letichevsky V, Ben-David J, Tal D, Tamir A, Talmon Y, Gordon CR, Luntz M (2004). Mal de debarquement and posture: reduced reliance on vestibular and visual cues. Laryngoscope.

[17] Cha Y, Cui Y (2013). Rocking dizziness and headache: a two-way street. Cephalalgia.

[18] Tal D, Wiener G, Shupak A (2014). Mal de debarquement, motion sickness and the effect of an artificial horizon. J Vestib Res.

[19] Clark BC, Leporte A, Clark S, Hoffman RL, Quick A, Wilson TE, Thomas JS (2013). Effects of persistent Mal de debarquement syndrome on balance, psychological traits, and motor cortex exctiability. J Clin Neurosci.

[20] MacKe A, LePorte A, Clark BC (2012). Social, societal, and economic burden of mal de debarquement syndrome. J Neurol.

[21] Arroll M, Attree E, Cha Y, Dancey C (2014). The relationship between symptom severity, stigma, illness intrusiveness and depression in Mal de Debarquement Syndrome.

[22] Pearce A, Davies C, Major B (2015) Efficacy of neurostimulation to treat symptoms of Mal de debarquement syndrome. A preliminary study using repetitive transcranial magnetic stimulation. J Neuropsychol 10.1111/jnp.12070 25809585

[23] Cha Y, Chakrapani S, Craig A, Baloh R (2012). Metabolic and functional connectivity changes in Mal de debarquement syndrome. PLoS One.

[24] Cha Y-H, Cui Y, Baloh RW (2013). Repetitive transcranial magnetic stimulation for mal de debarquement syndrome. Otol Neurotol.

[25] Hain T, Hanna P, Rheinberger M (1999). Mal de debarquement. Arch Otolaryngol Head Neck Surg.

[26] Shou G, Yuan H, Urbano D, Cha Y, Ding L (2014) Changes of symptom and EEG in mal de debarquement syndrome patients after repetitive transcranial magnetic stimulation over bilateral prefrontal cortex: a pilot study. In: Conference Proceeding of IEEE Engineering in Medicine and Biology Society, pp 4294–4297 10.1109/EMBC.2014.6944574 25570942

[27] Ding L, Shou G, Yuan H, Urbano D, Cha Y (2014). Lasting modulation effects of rTMS on neural activity and connectivity as revealed by resting-state EEG. IEEE Trans Biomed Eng.

[28] Dai M, Cohen B, Smouha E, Cho C (2014) Readaptation of the vestibulo-ocular reflex relieves the mal de debarquement syndrome. Front Neurol 5 10.3389/fneur.2014.00124 PMC4097942 25076935

[29] Dai M, Raphan T, Cohen B (2009). Adaptation of the angular vestibulo-ocular reflex to head movements in rotating frames of reference. Exp Brain Res.

[30] Guedry FJ, Graybiel A (1962). Compensatory nystagmus conditioned during adaptation to living in a rotating room. J Appl Physiol.

[31] Cha Y, Chakrapani S (2015) Voxel based morphometry alterations in Mal de debarquement syndrome. PLoS One 10(8) 10.1371/journal.pone.0135021 PMC4529307 26252893

[32] Jeong S-H, Jung K-Y, Kim J-M, Kim JS (2012). Medial temporal activation in Mal de debarquement syndrome revealed by standardized low-resolution brain electromagnetic tomography. J Clin Neurol.

[33] Clark B, Quick A (2011). Exploring the pathophysiology of mal de debarquement. J Neurol.

[34] Cha Y (2015). Mal de debarquement syndrome: new insights. Ann N Y Acad Sci.

[35] Nwagwu V, Patel R, Okudo J (2015) Mal de debarquement syndrome: a rare entity-a case report and review of the literature. Case Rep Otolaryngol 10.1155/2015/918475 PMC4540991 26346344

[36] Gibbs C, Commons K, Brown L, Blake D (2010). ‘Sea legs’: sharpened romberg test after 3 days on a live-aboard dive boat. Diving Hyperb Med.

[37] Cha Y, Cui Y, Baloh R (2013). Repetitive transcranial magnetic stimulation for mal de debarquement syndrome. Otol Neurotol.

[38] Ashburner J, Friston KJ (2000). Voxel-based morphometry—the methods. Neuroimage.

[39] Lempert T, Olesen J, Furman J, Waterston J, Seemungal B, Carey J, Bisdorff A, Versino M, Evers S, Newman-Toker D (2012). Vestibular migraine: diagnostic criteria. J Vestib Res.

[40] Graybiel A, Wood CH, Miller EF, Cramer D (1968) Diagnostic criteria for grading the severity of acute motion sickness, Pensacola 5648730

